# PSMA PET: the answer to MRI-occult prostate cancer… or another mirage?

**DOI:** 10.1007/s00330-026-12596-3

**Published:** 2026-04-30

**Authors:** Thomas Wagner, Mark Emberton, Iztok Caglic, Dimitris Priftakis, Francesco Giganti, Veeru Kasivisvanathan, Shonit Punwani

**Affiliations:** 1 Department of Nuclear Medicine, Royal Free London NHS Foundation Trust, London, UK; 2 Centre for Medical Imaging, Division of Medicine, University College London, London, UK; 3 Division of Surgery and Interventional Science, University College London, London, UK; 4 Department of Radiology, University College London Hospital NHS Foundation Trust, London, UK; 5 Institute of Nuclear Medicine, University College London Hospital NHS Foundation Trust, London, UK; 6 Centre for Urology Imaging, Prostate, AI and Surgical Studies (COMPASS) Research Group, Division of Surgery and Interventional Science, University College London, London, United Kingdom; 7 Department of Urology, Comprehensive Cancer Center, Medical University of Vienna, Vienna, Austria

**Keywords:** Prostate cancer, MRI, PSMA PET/CT

## Abstract

**Abstract:**

Multiparametric MRI (mpMRI) has transformed the diagnostic pathway for suspected prostate cancer by improving the detection of clinically significant prostate cancer (csPCa) and reducing unnecessary biopsies. However, its negative predictive value declines in higher-risk populations, and up to 30% of men with negative or indeterminate mpMRI (PI-RADS 1–3) may still harbour csPCa, particularly those with adverse clinical features. Variability in MRI quality, reader expertise, and reference standards further limits reproducibility outside expert centres. Prostate-specific membrane antigen (PSMA) PET-CT has emerged as a promising complementary modality. Beyond its role in staging and recurrence, prospective studies support its value in the pre-biopsy setting. Trials such as PRIMARY, PEDAL, and others demonstrate that PSMA PET/CT can detect a substantial proportion of csPCa missed by mpMRI, with high sensitivity and negative predictive value. The development of structured reporting systems, notably the PRIMARY score, represents an important step towards standardisation and improved comparability across studies. Despite encouraging diagnostic performance, critical uncertainties remain. The biological significance of PSMA-positive, MRI-negative lesions is unclear, and higher detection rates may partly reflect intensified sampling rather than clinically meaningful disease. Cost-effectiveness, optimal patient selection, and the positive predictive value of PSMA-avid lesions in MRI-negative prostates require further prospective validation. This opinion piece reviews high-quality prospective evidence and argues that PSMA PET/CT has the potential to refine risk stratification and reduce unnecessary biopsies in selected men with persistent suspicion of prostate cancer. Integration into diagnostic pathways should be guided by imaging performance and biological relevance, ensuring that the detected disease is clinically meaningful.

**Key Points:**

***Question*** *Can PSMA PET/CT improve risk stratification and reduce missed clinically significant prostate cancer in men with negative or indeterminate mpMRI?*

***Findings*** *Prospective studies show PSMA PET/CT complements mpMRI, achieving high sensitivity and negative predictive value while detecting a substantial proportion of significant cancers missed by MRI.*

***Clinical relevance*** *PSMA PET/CT may help safely avoid unnecessary biopsies and identify clinically significant disease in selected men after negative or equivocal MRI, but adoption requires standardised reporting, biological validation of PSMA-positive/MRI-negative lesions, and careful evaluation of cost-effectiveness within routine clinical pathways.*

**Graphical Abstract:**

**Figure:**
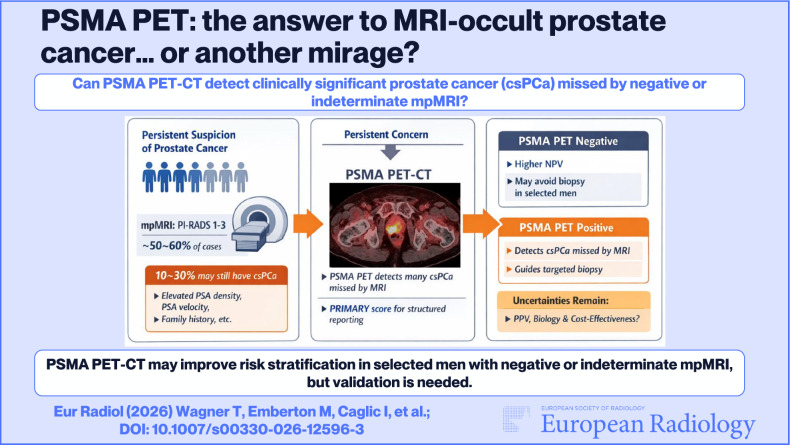

## Background

Multiparametric MRI (mpMRI) and its combination with MRI-targeted biopsy have revolutionised the diagnostic pathway of men with suspected prostate cancer (PCa) by improving the detection rate of clinically significant prostate cancer (csPCa), reducing the number of unnecessary biopsies and improving risk stratification in patients with low-risk PCa [1].

Despite these advantages, mpMRI has limitations. Although a meta‑analysis of > 9000 patients reported a negative predictive value (NPV) of 88% for csPCa, the NPV falls as disease prevalence rises, dropping to 67% when prevalence reaches 60% [2]. NICE guidance acknowledges that 11–28% of men with a ‘negative’ MRI may still harbour clinically significant cancer [3]. In routine practice, positive predictive value is also variable; a US multi‑centre study of 26 institutions found PI‑RADS 3–5 positive predictive values ranging from 18% to 70% [4].

In the PROMIS study, < 10% of patients with negative mpMRI had csPCa [5]. The apparent increase in the proportion of csPCa missed by mpMRI since PROMIS laid the ground for MRI-based triage is due to a combination of factors. The initial studies validating the role of MRI were performed in expert centres (high-quality MRI set-up and quality assurance, high-volume experienced MRI prostate radiologists and high-volume experienced urologists) with a robust histopathological standard (e.g., PROMIS performed systematic template mapping biopsies every 5 mm, which were reported by an expert uropathologist). These multiple layers of expertise in the setting of a clinical trial led to results that may not reproduce or scale to routine clinical practice in a variety of hospital settings. MRI quality is highly variable between centres, and radiologists’ PI-RADS scoring is inconsistent 20–25% of the time [6]. More recent studies published since PROMIS and PRECISION [7] have used a composite reference standard for the presence of csPCa, which is probably not as robust as the reference standard from PROMIS and PRECISION.

There remains controversy on the optimal management for patients with a negative or indeterminate mpMRI, with varying practice between experienced centres. Biomarkers such as PSA density and PSA velocity provide additional information on the probability of the presence of csPCa and are used clinically for risk stratification [1]. The European Association of Urology (EAU) guidelines recommend risk-adapted biopsy decision by incorporating PSA density to improve PPV and NPV of MRI [8]. In addition, NICE recommends repeat PSA for men with a strong suspicion of prostate cancer despite negative mpMRI (e.g., PSA density > 15 ng/mL/mL or PSA velocity > 75 ng/mL/year or strong family history) [3] Besides PSA density, Prostate-specific membrane antigen (PSMA) PET-CT seems another promising solution to improve the diagnostic performance of MRI.

PSMA is a transmembrane glycoprotein that is overexpressed in prostate cancer; the degree of PSMA expression in prostate cancer correlates with Gleason score and tumour aggressiveness [9]. When labelled with ^68^Ga or ^18^F, PSMA emits positrons whose annihilation reaction can be detected with PET-CT.

**Fig. 1:**
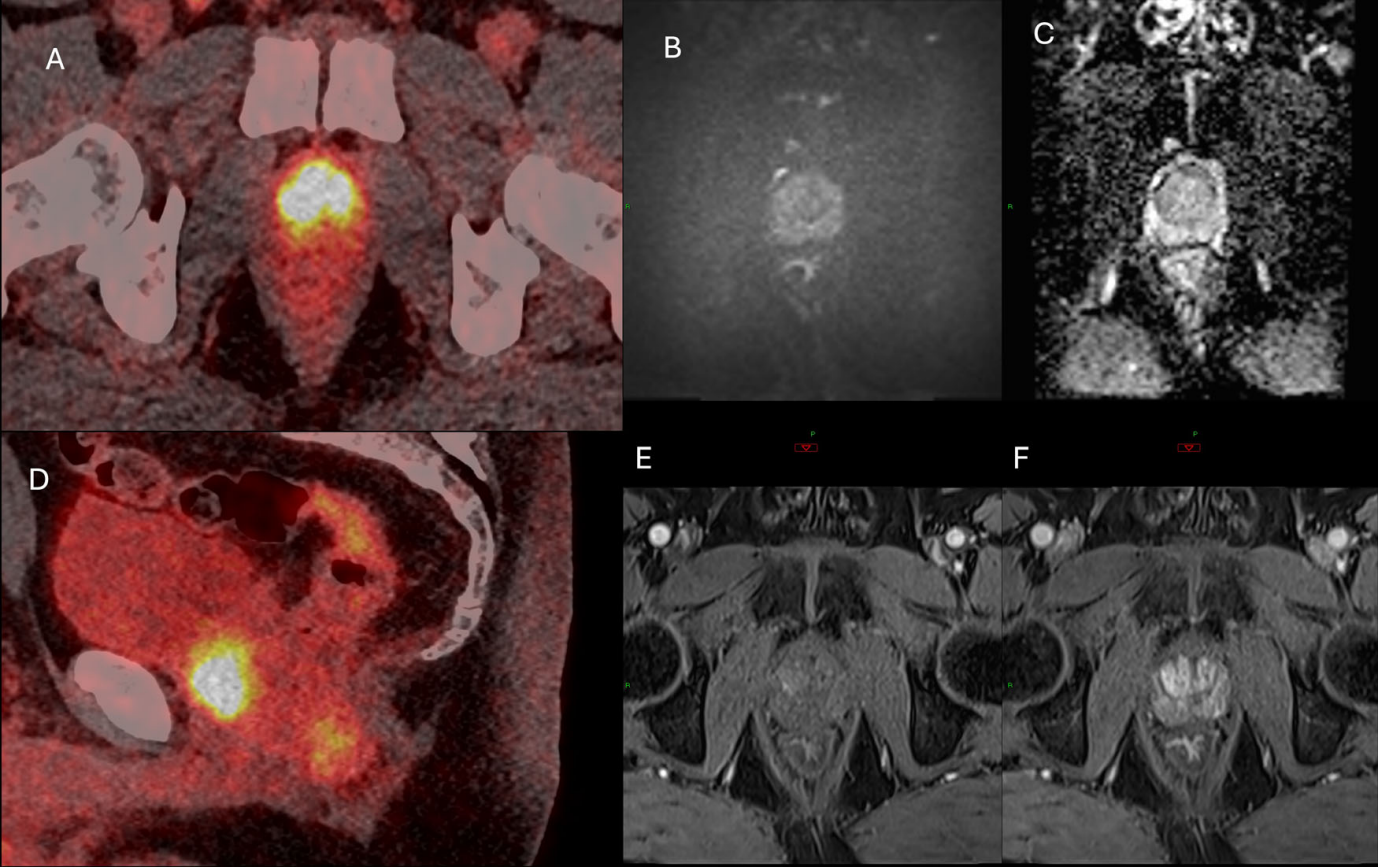
MRI-negative and PSMA-positive prostate malignancy. 56-year-old patient with PSA 11.1 ng/mL, PSA density 0.38, prostate 28 mL. MRI report of PI-RADS 2. Biopsy showed Gleason 3 + 4 prostate malignancy. PSMA PET/CT demonstrated a 3.6 cm intensely PSMA-avid area in the apex of the prostate and in the transitional zone, SUVmax 13.9. Intensely PSMA-avid prostate malignancy centred in the transitional zone. **A**, **D** (Fused transverse and sagittal) PSMA PET/CT. Negative multiparametric prostate MRI, homogeneous gadolinium enhancement pattern (**E**, **F**), axial diffusion-weighted imaging b1400 (**B**) and axial apparent diffusion coefficient map (**C**) showed no MRI-visible lesion corresponding to the PSMA-avid lesion

PSMA PET/CT has already redefined the landscape for imaging prostate cancer. It is routinely used globally for recurrence detection [10] and is increasingly adopted for high-risk staging for nodal and distant metastases [11]. PSMA PET might be a useful adjunct to MRI for T staging: a meta-analysis showed higher sensitivity and similarly high specificity for PSMA-PET/MR than MRI to detect extracapsular extension and seminal vesicle involvement [12]. More recent studies have been focusing on the role of PSMA PET/CT in the pre-biopsy diagnostic pathway as a complement to mpMRI. Notably, the PRIMARY trial demonstrated that the combination of PSMA PET/CT and mpMRI performed better than mpMRI alone, with an improvement in NPV (91% vs 72%) and sensitivity (97% vs 83%) with preserved positive predictive value (PPV) [13]. In our clinical practice, we have also encountered anecdotal evidence of PSMA-positive csPCa in patients with negative MRI (Fig. 1).

PSMA PET/CT has the potential to mitigate the risk of missing csPCa on mpMRI, and its integration in the pre-biopsy diagnostic pathway offers an interesting option for the management of men with negative or indeterminate mpMRI and persistent suspicion of prostate cancer.

While the diagnostic yield of mpMRI and PSMA PET is often discussed in imaging terms, it is important to acknowledge the biological dimension underpinning visibility. MRI-visible lesions typically represent tumour foci that have achieved sufficient cellular mass, neovascularisation and stromal remodelling to be detectable on anatomical and diffusion-weighted sequences. In contrast, MRI-invisible foci may represent smaller, biologically less advanced clones that have not yet acquired these hallmarks of malignant progression [14, 15].

This review will focus on high-quality evidence from prospective studies evaluating PSMA PET in men with negative or indeterminate mpMRI.

**Table 1:** Summary of prospective studies investigating PSMA PET in patients with PI-RADS 1–3

Article	Scan	Tracer	Performance of PSMA PET in PI-RADS 2 and 3 lesions	PSMA reference and positivity	Biopsy method and definition of csPCa	Reader experience	Inclusion criteria	Blinding between MRI and PSMA
Emmett et al [13]	Pelvic PET-CT	^68^Ga-PSMA-11	28% (27/95) of PI-RADS 2 had csPCA 47% (25/53) of PI-RADS 3 had csPCa 90% sensitivity of PSMA PET-CT in PI-RADS 2–3 lesions (36% (52/146) patients with PI-RADS 1–3 lesions had csPCa)	Four-quadrant model and SUVmax per quadrant. Negative if SUVmax < 4. Positive if SUVmax > 4 and four-point certainty scale probably or definitely positive	Systematic transperineal biopsies with a minimum of 18 cores. Additional targeted biopsies for MRI and PSMA-positive lesions. CsPCA if ≥ ISUP 2	PI-RADS v2, positive if PI-RADS 3–5 MRI locally reported, no detail on reader experience	Raised PSA < 20 ng/mL or abnormal DRE Exclusion: previous prostate cancer, biopsy or MRI	Yes
Shi et al [20]	Whole body PET-MRI and pelvic MRI	^68^Ga-PSMA-11	sensitivity, specificity, PPV and NPV of 87.5%, 83.3%, 46.7% and 97.5% for detecting csPCa in PI-RADS 3 patients (14% (8/56) patients had csPCa)	PRIMARY score 4–5 defined as positive, SUVmax in each prostate quadrant	Transperineal 12-core systematic biopsy and mpMRI/ultrasound fusion-guided targeted biopsy CsPCA if ISUP > 1	2 readers with > 10 years of experience and uroradiology subspecialty, PI-RADS v2 Concordant findings from 2 readers	Biopsy-naive men with single or multiple PI-RADS 3 lesions on mpMRI	Not mentioned, but PSMA PET-MR has a pelvic MRI
Wong et al [21]	PET-CT thoracic inlet to thighs	^18^F-DCFPyL	Sensitivity 82% for csPCa in PI-RADS 1–2 patients (18% (11/61) of PI-RADS 1–2 patients had csPCa) Sensitivity 33% for csPCa in PI-RADS 3 patients (38% (6/16) of PI-RADS 3 patients had csPCa)	SUVmax < 4 negative, 4–7 equivocal and > 7 positive. Qualitative reporting using a 5-point scale and reader’s expertise (10, 25, 50, 75 and 90% certainty scores). PSMA positivity if either SUVmax > 7 and certainty score ≥ 4. Location of findings described with PI-RADS v2.1 sector map.	Targeted (min of 4 cores) and systematic biopsy (min of 18 cores). CsPCA if ≥ ISUP 2	High-volume subspecialist prostate MRI radiologists. PI-RADS v2.1. Positive MRI if PI-RADS ≥ 3	Abnormal DRE, raised PSA or free/total PSA ratio < 25%, family history. Exclusion if previous PCa, biopsy or mpMRI, other active malignancy, hip replacement or CI to MRI	Yes
Ferraro et al [22]	Pelvic PET-MRI	^68^Ga-PSMA-11	Sensitivity 100% for csPCa in PI-RADS 3 (14%) (1/7) PI-RADS 3 patient had csPCa	Not specified	PSMA PET-MRI targeted biopsy and section-based saturation template biopsy. CsPCa if ISUP ≥ 3 or core length ≥ 6 mm	2 readers, > 10 years and > 5 years experience	Elevated PSA (> 2.5 ng/mL for 30–50 years and > 4 ng/mL for 50–80 years) and PI-RADS ≥ 3. Exclusion criteria were age < 30 and > 80, previous biopsy within 8 weeks prior to imaging, previous pelvic irradiation, prostatectomy, transurethral resection of the prostate (TURP) or androgen deprivation hormonal therapy (ADT), and any contraindication to MRI or prostate biopsy as well active urinary tract infection or indwelling catheter.	Not mentioned, but PSMA PET-MRI (not detailed if MRI included prostate MRI)
Prive et al [25]	Vertex to thighs	18F-PSMA-1007	For PI-RADS 3 lesions, NPV 93% and PPV 27%	Level of suspicion 5-point scale based on reader experience	Targeted biopsy with MIR in-bore or MRI/transrectal US fusion. CsPCA if ≥ ISUP 2	Three uroradiologists with > 10 years of experience with PI-RADS	Raised PSA and/or abnormal DRE. Exclusion criteria: history of prostate cancer, biopsy in the last 6 months or history of pelvic surgery that could impair image interpretation	No blinding, PSMA readers aware of PI-RADS scores

### Prospective studies review (Table 1)

The PRIMARY trial [13] recruited 296 men with suspected prostate cancer; 291 men had mpMRI, pelvic PSMA PET/CT and systematic plus targeted biopsy. 28% (27/95) of men with PI-RADS 2 and 47% (25/53) of men with PI-RADS 3 had csPCa. PSMA PET/CT was positive in 90% (47/52) of these cancers. The rate of patients with PI-RADS 1–3 was 50% (148/296).

PSMA PET/CT detected 90% of csPCa in patients with negative or indeterminate mpMRI, raising the possibility of offering PSMA PET/CT as a second-line investigation in selected men.

The PRIMARY score is a 5-point scale which was developed using data from the PRIMARY trial to standardise assessment of uptake in the prostate based on location, patterns and intensity of uptake [16]. This is a great development as most other studies evaluating PSMA PET/CT for the diagnosis of prostate cancer relied on variable standards, usually based on a combination of intensity of uptake measured as SUVmax or ratio of SUVs and experience of the reporter regarding the pattern and location of uptake. The PRIMARY score provides a useful template for standardising future research and has been validated on external cohorts [17, 18].

The same team is currently running the PRIMARY 2 trial [19], which is recruiting 660 men with either PI-RADS 2 and high-risk features or PI-RADS 3 and randomising them to pelvic PSMA PET/CT-guided biopsy or template transperineal prostate biopsy. In the intervention arm, PSMA uptake will be evaluated using the PRIMARY score (PRIMARY scores 1–2 considered as negative and 3–5 as positive). Patients with a positive PSMA PET/CT will undergo targeted transperineal biopsies of up to 4 PSMA-positive lesions. Patients with a negative PSMA PET/CT will omit biopsy and be followed up for 2 years. The objectives of the trial are to estimate the difference in csPCa between the control and experimental arms and the percentage of men avoiding prostate biopsy in the experimental arm.

Shi et al prospectively assessed 56 biopsy-naive men with PI-RADS 3 with ^68^Ga-PET/MRI and the PRIMARY score (with scores 4–5 classified as positive) [20]. 14% (8/56) patients had csPCa. PSMA PET/CT had sensitivity, specificity, PPV and NPV of 87.5%, 83.3%, 46.7% and 97.5% for detecting csPCa.

The PEDAL study [21] recruited 236 patients with suspected prostate cancer and scanned them with mpMRI (positive for PI-RADS 3–5) and F-DCFPyL PSMA PET/CT (positive if SUVmax > 7, equivocal for SUVmax 4–7 and also using a 5-point certainty score based on the experience of the reporter). Patients with positive findings on either modality had a biopsy. 184 patients had a biopsy, 84 (45.6%) had csPCa. mpMRI had higher diagnostic accuracy than PSMA PET/CT for detecting PCa and for csPCa (although not clinically significant). Of the 61/184 (33%) patients with PI-RADS 1–2, 11/61 (18%) had csPCa, with 9/11 (82%) positive on PSMA PET/CT.

Of the 16/184 (9%) patients with PI-RADS 3, 6/16 (38%) had csPCa, with 2/6 (33%) positive on PSMA PET/CT.

Overall, PSMA PET/CT detected 11/17 (65%) of csPCa in patients with PI-RADS 1–3 mpMRI.

Ferraro et al recruited 42 patients with suspected prostate cancer and PI-RADS ≥ 3 on mpMRI; patients had ^68^Ga-PSMA11 PET-MRI and targeted as well as systematic biopsies [22]. PSMA PET-MRI had very good diagnostic performance with sensitivity, specificity, PPV and NPV of 96%, 81%, 89% and 93% with accuracy of 90%.

Seven patients had PI-RADS 3 lesions, 1/7 (14%) had csPCa and was positive on PSMA PET-MRI.

Lopci et al recruited 97 patients with either negative mpMRI (PI-RADS ≤ 2) or positive mpMRI and negative biopsy or contraindications to MRI. 64 patients (66%) had positive PSMA PET/CT findings (focal uptake greater than background activity) and underwent PET-TRUS fusion-guided biopsy [23]. 15/64 patients (23%) had csPCa. Of 15 patients with negative mpMRI, 4/15 (26.7%) had csPCa identified as a result of ^68^Ga-PSMA PET-TRUS fusion-guided biopsy.

The same team are currently recruiting for the PROSPET-BX study [24], to assess the diagnostic performance of Ga-PSMA PET/CT—TRUS fusion biopsy compared to mpMRI—TRUS fusion biopsy. 128 patients will be recruited and undergo mpMRI and Ga-PSMA PET/CT. Positive lesions on mpMRI and PSMA PET/CT will be biopsied. Patients with negative PSMA PET/CT and mpMRI will have systematic biopsies.

Prive et al recruited 75 patients with suspicion of prostate cancer who underwent both mpMRI and PSMA PET/CT with ^18^F-PSMA-1007 [25]. PSMA scans were scored using a 5-point scale (level of suspicion LOS) based on reader experience. Patients with PI-RADS ≥ 3 or LOS ≥ 3 had targeted biopsies (except for LOS 3 that were clearly benign on MRI). Patients with PI-RADS 1–2 and LOS 1–2 had PSA monitoring for 12 months with repeat MRI if rise > 25%. Of the 26 patients with PI-RADS 3 lesions, 3 (12%) were upgraded and 14 (54%) were downgraded after PSMA PET/CT, with an NPV of 93% and PPV of 27% to detect csPCa.

## Discussion

50–60% of men with suspected prostate cancer have an mpMRI with PI-RADS 1–3, representing negative or indeterminate probability of csPCa [5, 7]. 10–30% of these patients will have csPCa [3, 5] with a higher prevalence of csPCa in the subgroup of patients with high-risk features such as BCRA mutation, raised PSA density or PSA velocity and strong family history. However, whether these MRI-occult lesions represent biologically significant disease remains uncertain. MRI visibility may itself be a biomarker of biological viability—reflecting a threshold of tumour mass, angiogenesis, and genetic heterogeneity required for progression [15].

Furthermore, long-term data from active surveillance cohorts, including the Memorial Sloan Kettering series, suggest negligible prostate cancer–specific mortality at 15 years among men with low-risk tumours [26]. Similarly, molecular profiling studies indicate that MRI-visible lesions are enriched for mutations associated with progression and lethality, whereas MRI-invisible foci lack these alterations [27]. Molecular profiling and genetic markers data are not yet known for PSMA-avid lesions, although it is likely that they will be similar to MRI-visible lesions, as most csPCa lesions are both MRI-visible and PSMA-avid. Genetic and molecular profiling data on PSMA-avid/MRI-invisible would be of great interest to better understand their risk profile.

PSMA PET/CT has shown its ability to detect csPCa that is missed by mpMRI, but the biological significance of these PSMA-positive lesions remains uncertain.

Across the prospective studies summarised in Table 1, PSMA PET showed encouraging diagnostic performance in men with negative or indeterminate mpMRI, with generally high sensitivity and negative predictive value for clinically significant prostate cancer. However, direct comparison across studies remains difficult because of heterogeneity in patient selection, MRI categories included, PSMA interpretation criteria, reader experience, and biopsy reference standards. The key overall finding is that PSMA PET appears complementary to mpMRI in selected patients, but the magnitude of benefit and optimal role within diagnostic pathways remain to be defined prospectively [13, 20–22]. The overall sensitivity of PSMA PET/CT to detect csPCa in patients with negative or indeterminate mpMRI across these 4 studies is impressive and lays the ground for PSMA-guided diagnostic pathways for patients with persistent suspicion of prostate cancer after negative or indeterminate mpMRI.

There are limitations to these studies. Most use different criteria for PSMA PET positivity. Positive lesions are usually based on a combination of SUVmax, uptake greater than background activity, focality and location of the uptake. The pattern of uptake seems important, with multiple studies using the reporter’s experience to assess the probability of malignancy with Likert-style scales. A great effort by Emmett et al was the development and subsequent validation of a 5-point scale of various patterns of PSMA uptake and correlating it to the probability of malignancy, the PRIMARY score [16]. This score was validated externally [17, 18] and is already in use in ongoing prospective research trials [19]. The use of the PRIMARY score will provide needed standardisation in future research trials, will allow easier comparison across protocols and has the potential to improve the diagnostic performance of PSMA PET/CT. It could eventually translate into a useful clinical tool.

Beyond standardising PSMA PET interpretation alone, recent work has sought to integrate mpMRI and PSMA PET into a single composite model. The 5-point P score, which combines PI-RADS and PRIMARY scores, improved diagnostic accuracy for clinically significant prostate cancer over either modality alone in its initial external validation. Although not yet sufficiently validated for routine pathway decisions, especially in the MRI-negative or equivocal subgroup that is the focus of this review, it is an important step towards a standardised multimodality framework that could ultimately support biopsy selection and imaging-directed diagnostic pathways [28].

Most of the studies use a combination of targeted and systematic prostate biopsies and similar definitions for csPCa, which are therefore unlikely to explain the heterogeneous findings across the studies.

A striking/surprising finding of the PRIMARY trial was the high prevalence of csPCa in patients with PI-RADS 1–3 (28% of PI-RADS 2, 47% of PI-RADS 3 and 35% of all patients with PI-RADS 2–3) [13]. This is higher than expected and likely due to the recruitment of patients with clinical red flags but negative or equivocal MRIs. In the PEDAL study, the prevalence of csPCA in PI-RADS 2 was 18% and 38% in PI-RADS 3 [21]. In Shi et al’s study, 14% of patients with PI-RADS 3 had csPCa [20].

These results are at odds with mpMRI studies such as PROMIS. Robust prospective studies are needed to shed light on the prevalence of csPCa in patients with negative or indeterminate mpMRI.

The emerging question is whether PSMA-positive but MRI-negative lesions share the molecular and phenotypic attributes of MRI-visible tumours, or whether they reflect early, less advanced disease. PSMA overexpression correlates with tumour aggressiveness, yet it may occur in the absence of sufficient neovascular or stromal change to render the lesion visible on MRI. Determining whether PSMA-positive/MRI-negative foci represent a biologically aggressive subset that will later become MRI-visible is critical to defining the role of PSMA PET in this population. An alternative explanation is of suboptimal image quality or reading expertise in prostate MRI, and that the MRI-invisible lesions highlighted by PSMA PET/CT would turn visible in expert centres with high-image quality and reader expertise.

An additional confounder is sampling intensity. Random or PSMA-targeted biopsies inherently increase the likelihood of finding small pattern-4 foci that meet the histological definition of csPCa without necessarily indicating clinically meaningful disease. The higher detection rates in PSMA-guided protocols may therefore partly reflect enhanced sampling rather than true biological difference.

Clinically significant prostate cancer is a heterogeneous category rather than a single disease state, encompassing small-volume Gleason 3 + 4 lesions as well as large-volume Gleason 4-predominant or Gleason 5 tumours with very different prognostic significance. This distinction is particularly important when evaluating PSMA-guided biopsy pathways, as increased sampling of PSMA-avid lesions may preferentially identify small foci that satisfy histological criteria for clinically significant disease without necessarily representing the same level of biological or clinical risk. Future trials should therefore examine not only overall csPCa detection, but also the grade, volume, imaging phenotype, and downstream clinical relevance of the cancers detected.

The PRIMARY 2 trial will provide important data on the PSMA PET/CT-guided diagnostic pathway, randomising patients with PI-RADS 2 and high-risk features or PI-RADS 3 to PSMA PET/CT-guided biopsy or template perineal prostate biopsy [19]. The trial is designed to demonstrate the percentage of patients avoiding biopsy in the PMSA-guided arm and the difference in csPCa in the control and experimental arms.

There remain important questions that prospective trials investigating PSMA-targeted biopsy will need to elucidate before we can move on to designing clinical pathways making the best use of PSMA PET/CT’s capabilities.

While the PRIMARY 2 trial will be important in demonstrating how PSMA PET can reduce the burden of unnecessary biopsies, this addresses only one part of the diagnostic pathway. The next critical question is not simply how many men can avoid biopsy, but rather what it means when PSMA PET is positive in a prostate that appears negative or indeterminate on MRI. Focal uptake could represent clinically significant cancer, indolent disease or a benign finding, and without prospective validation, we cannot assume it should guide targeted biopsy. Establishing the PPV of PSMA-avid lesions in this setting is therefore essential. Such data would provide the foundation for safely integrating PSMA-targeted biopsy into routine practice and ensure that we are not only sparing men from unnecessary procedures but also accurately identifying those who truly need intervention.

The cost-effectiveness of PSMA PET/CT, including diagnostic pathways, will need to be investigated. PSMA PET/CT is an expensive test, especially in the UK, where it costs 2.5–6 times more than in Australia [29]. A study from the Netherlands showed that PSMA PET/CT may be cost-effective in patients with PI-RADS 3; however, at a high incremental cost-effectiveness ratio, which is not acceptable to NICE, and at a cost that is inferior to the one available in the UK [30]. It was not cost-effective in patients with PI-RADS 1–2. In addition, cost-effectiveness data are inherently healthcare system-dependent, which may limit their generalisability.

The biological significance of PSMA overexpression and the extent to which PSMA positivity reflects clinically meaningful aggressive disease remain incompletely understood. Importantly, the complementarity between mpMRI and PSMA PET is bidirectional rather than limited to improved sensitivity after MRI: pathology-correlated studies show that discordance between the two modalities is common, and lesions detected on only one modality may still represent clinically significant cancer [31]. In addition, PSMA-positive/MRI-negative tumours appear smaller than lesions visible on both modalities, suggesting that discordance may reflect differences in tumour phenotype rather than simple test failure [31]. Emerging radioproteomic data provide further support for this concept, showing that PSMA PET and mpMRI features correlate with distinct proteomic programmes within localised prostate cancer, with PSMA-derived metrics demonstrating broader and more functionally diverse protein associations than mpMRI features. In particular, PSMA-associated features were linked to pathways related to tumour heterogeneity and metabolic activity, raising the possibility that combined PSMA/mpMRI phenotypes may reflect biologically distinct states [32, 33]. These observations remain preliminary, but they provide a useful framework for understanding why MRI and PSMA PET may reveal overlapping yet non-identical aspects of clinically significant disease.

The last two decades have brought dramatic changes to how we manage patients with prostate cancer. One of the major trends has been to treat fewer patients and to risk-stratify treatments based on histopathological markers. Whereas all patients were offered radical prostatectomy not so long ago, now only patients with Gleason 3 + 4 and higher-risk disease are offered treatment, with focal treatment and strategies of active surveillance with repeat PSA and mpMRI being offered. A similar theme is observed in deciding which patients to biopsy; only men with high-risk features and PI-RADS 2 are being offered biopsy. Some men with PI-RADS 3 are followed up with PSA surveillance.

The other major trend has been towards non-invasive characterisation of disease: mpMRI and MRI-based decision to biopsy pathways revolutionised our understanding of prostate cancer and what we call significant disease. However, no long-term prognostication has been performed to evaluate what happens at the population level in the era of MRI-based selection of men for prostate biopsy.

PSMA PET/CT is emerging as a potential new imaging modality to further risk-stratify selected patient groups. Its main utility may be in reducing unnecessary biopsies, as it seems complementary to MRI in ruling out csPCa. However, we may observe a similar reduction in diagnostic accuracy and utility as PSMA PET goes from carefully selected research patient populations in expert centres to routine clinical practice.

Future research should integrate molecular, volumetric and imaging data to determine whether MRI-negative/PSMA-positive lesions share the same biological landscape as MRI-visible tumours. Longitudinal imaging could test whether such lesions evolve into MRI-visible disease over time, and histopathological correlation should assess tumour burden concordance, core length, and clonal similarity. These insights will be pivotal to define whether PSMA PET is revealing truly significant disease or detecting indolent clones.

We remain optimistic that future advancements will identify the appropriate sequence of tests to carefully select the patients needing treatment while avoiding harm to those who can be safely monitored.

## Conclusion

PSMA PET/CT shows considerable promise as a complementary test for men with negative or indeterminate mpMRI yet persistent suspicion of prostate cancer. Prospective data indicate high sensitivity and NPV, suggesting that a PSMA‑guided pathway could safely reduce unnecessary biopsies while enhancing detection of clinically significant disease, but these findings need to be confirmed by ongoing prospective trials. Ultimately, the integration of PSMA PET into diagnostic pathways should be guided not only by imaging performance but also by biological understanding—ensuring that what we detect represents disease that matters.

## Abbreviations

csPCa: Clinically significant prostate cancer
EAU: European Association of Urology
mpMRI: Multiparametric MRI
NPV: Negative predictive value
PPV: Positive predictive value
PSMA: Prostate-specific membrane antigen
